# Therapeutic Potential of Different Natural Products for the Treatment of Alzheimer's Disease

**DOI:** 10.1155/2022/6873874

**Published:** 2022-07-22

**Authors:** Biswajit Chakraborty, Nobendu Mukerjee, Swastika Maitra, Mehrukh Zehravi, Dattatreya Mukherjee, Arabinda Ghosh, Ehab El Sayed Massoud, Md. Habibur Rahman

**Affiliations:** ^1^Department of Biochemistry and Biophysics, University of Kalyani, Nadia, West Bengal, India; ^2^Department of Microbiology, Ramakrishna Mission Vivekananda Centenary College, West Bengal, India; ^3^Department of Health Sciences, Novel Global Community Educational Foundation, Australia; ^4^Department of Microbiology, Adamas University, India; ^5^Department of Clinical Pharmacy Girls Section, Prince Sattam Bin Abdulaziz University, Al-Kharj, Saudi Arabia; ^6^Department of Microbiology, Jinan University, China; ^7^Microbiology Division, Department of Botany, Gauhati University, Guwahati, Assam 781014, India; ^8^Biology Department, Faculty of Science and Arts in Dhahran Al Janub, King Khalid University, Abha, Saudi Arabia; ^9^Research Center for Advanced Materials Science (RCAMS), King Khalid University, Abha, Saudi Arabia; ^10^Agriculture Research Centre, Soil, Water and Environment Research Institute, Giza, Egypt; ^11^Department of Pharmacy, Southeast University, Banani, 1213 Dhaka, Bangladesh

## Abstract

A high incidence of dementia (60–80%) and a high rate of memory loss are two of the most common symptoms of Alzheimer's disease (AD), which affects the elderly. Researchers have recommended that traditional Chinese medicine (TCM) and Indian medicines can be used to prevent and cure AD. Several studies have linked neuroinflammation linked to amyloid-*β* (A*β*) deposition in the brain to the pathophysiology of neurodegenerative disorders. As a result, more research is needed to determine the role of inflammation in neurodegeneration. Increased microglial activation, cytokine production, reactive oxygen species (ROS), and nuclear factor kappa B (NF-*κ*B) all play a role in the inflammatory process of AD. This review focuses on the role of neuroinflammation in neuroprotection and the molecular processes used by diverse natural substances, phytochemicals, and herbal formulations in distinct signaling pathways. Currently, researchers are focusing on pharmacologically active natural compounds with the anti-neuroinflammatory potential, making them a possible contender for treating AD. Furthermore, the researchers investigated the limits of past studies on TCM, Indian Ayurveda, and AD. Numerous studies have been carried out to examine the effects of medicinal whole-plant extracts on AD. Clinical investigations have shown that lignans, flavonoids, tannins, polyphenols, triterpenoids, sterols, and alkaloids have anti-inflammatory, antiamyloidogenic, anticholinesterase, and antioxidant properties. This review summarizes information about numerous medicinal plants and isolated compounds used in the treatment of AD and a list of further references.

## 1. Introduction

AD is a prevalent neurological condition that is associated with a high risk of dementia (60–80%) [1]. Degenerative disorders of the central nervous system are characterized by alterations in the central nervous system that have severe psychological and physiological implications. AD, a neurodegenerative ailment of great significance and the leading cause of dementia in the elderly population, is now officially recognized. Under a microscope, AD is distinguished by the presence of intraneuronal neurofibrillary tangles (NFTs) and extracellular senile plaques (also known as amyloid plaques) in the brain. NFTs are formed because of a typical tau protein deposition and hyperphosphorylation, whereas senile plaques are formed because of extracellular deposits of A*β* peptides deposited in the brain. It is estimated that according to the Centers for Disease Control and Prevention (CDC), AD is the sixth greatest cause of death in the U.S.A. According to current projections, 5.4 million people in the U.S.A will be affected by AD by the middle of this century, with a total of 15 million estimated by the end of the century [1]. The diagnosis of AD happens once every 66 seconds [2], and 44 million people worldwide are affected by the disease or related dementia because of the condition. However, while the current projections predict that this ratio would more than treble by 2050 [3], this is not a foregone conclusion. There are three ways that AD expresses itself: memory loss, cognitive impairment, and eventually, the inability to care for oneself. As a result of this development [4, 5], AD units in nursing homes have risen to become the most common type of the special care unit in the institution. Caring for AD patients not only puts a financial strain on the family but also puts a mental strain on everyone who is involved in the process. As a result of this condition, the prevention and treatment of AD pose a significant challenge to public health and medical systems. As a result, significant funding should be allocated to basic research into the condition. A substantial amount of money has been put into the study and development of AD treatments over the last three decades. As of right now, there are no Food and Drug Administration- (FDA-) approved treatments for the condition. The FDA has only approved clinical medications for the treatment of AD so far [5]. Among the acetylcholinesterase inhibitors [5] that have been discovered are tacrine, donepezil, rivastigmine, and galantamine, to name a few. However, even though the drugs' therapeutic potential was demonstrated, the unpleasant side effects of nausea, vomiting, diarrhea, sleeplessness, and a slowed heart rate could not be overlooked. Mantine is yet another medicine that acts by suppressing the glutamate neurotransmitter in the brain, as above described. In AD patients, these treatments may be able to slow the pace of their memory loss but it is unlikely that they will be able to reverse it entirely. Therefore, AD is an extremely challenging condition that cannot be properly treated by concentrating just on a single treatment target.

**Figure 1:**
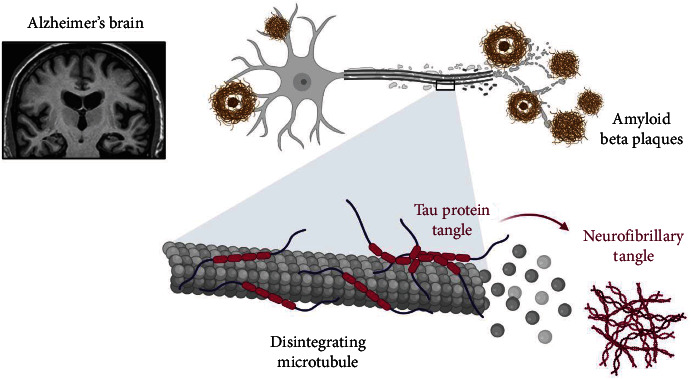
Disintegrating microtubules in AD (made with BioRender).

In AD and other tauopathies, tau, a microtubule-associated protein, develops insoluble filaments that band together to create neurofibrillary tangles. Under physiological circumstances, tau controls the assembling and structural stability of microtubules. The microtubules break down, and the liberated tau molecules organize form-paired helical filaments in the sick brain, where tau is abnormally hyperphosphorylated. Numerous pieces of evidence point to a malfunction in cellular signaling, specifically an imbalance in the activity of different protein kinases and phosphatases, as the root cause of tau hyperphosphorylation. It suggests that a critical factor in the imbalance in Alzheimer's disease is the *β*-amyloid peptide (A*β*) (as shown in Figure 1) [6].

More than 3000 years have passed since traditional Indian and Chinese medicine was first utilized in China and other South Asian nations to both prevent and cure neurodegenerative disorders [7, 8]. A fundamental principle of TCM is the concept of comprehensive treatment, which lies at the heart of the practice. Currently, it is mostly utilized in the form of multitarget and multichannel treatment, which can be used to prevent and treat diseases that have several targets and are difficult to treat, such as cancer. More recently, several studies have demonstrated that a variety of herbs and components derived from herbs are effective against AD while causing fewer adverse effects than traditional drugs, according to the findings.

**Figure 2:**
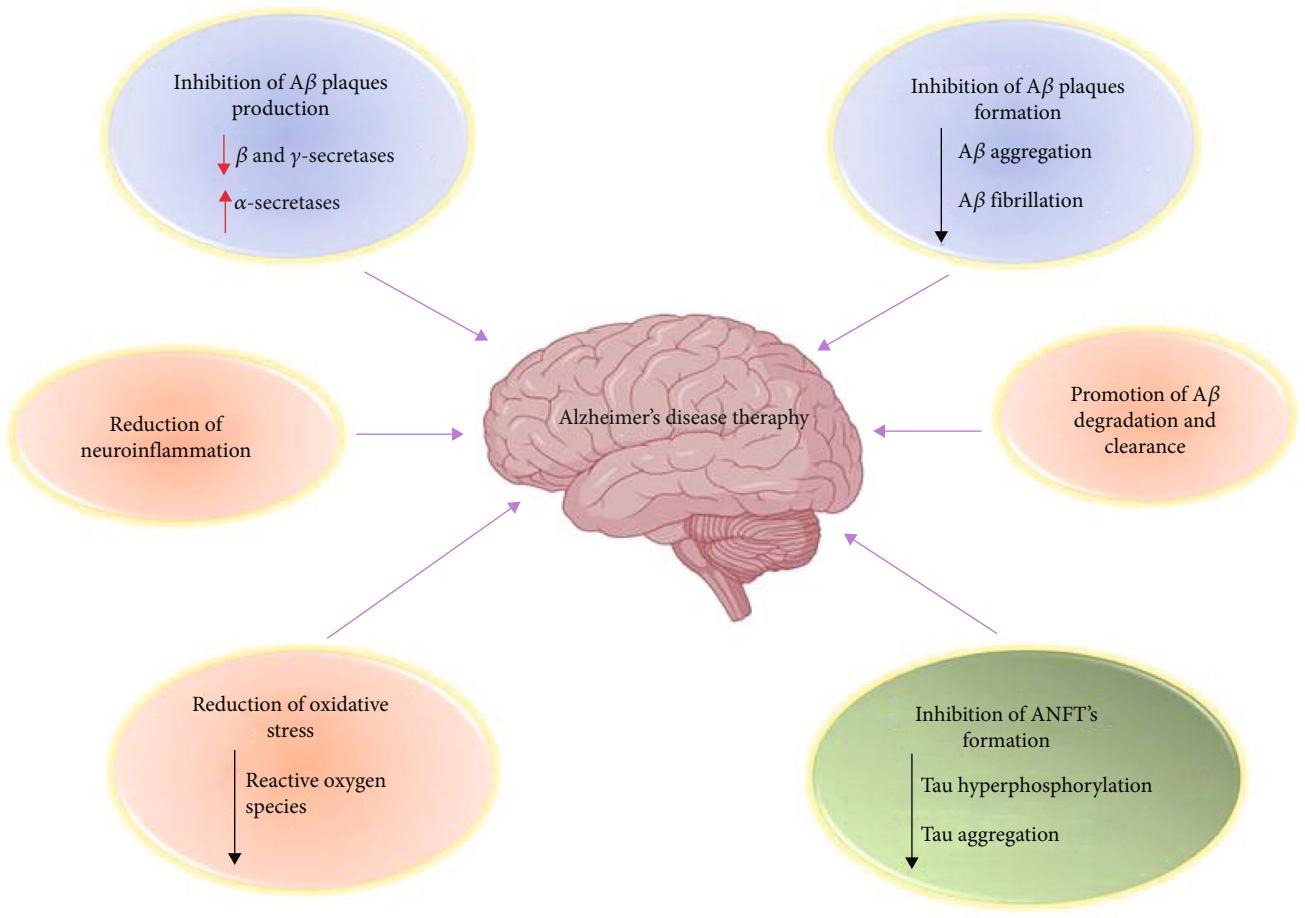
The many pathways linked with Alzheimer's disease (AD) therapy are depicted schematically. The decline and increase of the phenomenon are indicated by arrows pointing down and up, respectively (made with BioRender).

As a result of these discoveries, a new AD treatment strategy is required. Natural substances were the first molecules to be employed as medicinal agents, and they are still used today. Natural chemicals are currently being studied for their potential neuroprotective properties, piquing the interest of both science and industry. A wide range of natural compounds derived from various sources is effective in the prevention and treatment of a few diseases, including neurological disorders like AD. Natural compounds have been shown to have therapeutic potential in various in vitro and in vivo experiments, but only a small number of these molecules have moved to clinical trials. Natural chemicals' disease-preventive actions can be connected to a variety of pathways (as shown in Figure 2) [9].

Researchers have proven that *Ginkgo biloba* extracts, such as EGB761, can improve cognitive function, neuropsychiatric symptoms, and functional capacities in AD sufferers. In herbal treatments, baicalein [10], tanshinone [11], and huperzine A are monomers that have been proven to have therapeutic effects against AD. This review is aimed at summarizing information about numerous medicinal plants and isolated compounds used in the treatment of AD.

## 2. The Pathology and Pathophysiology of AD

The A*β* theory and the tau protein hypothesis, two of the most widely accepted hypotheses, both argue that A*β* plaques in the brain cause AD [12]. The A*β* theory is the most well acknowledged and commonly used of the three possible scenarios. The A*β* hypothesis is the most largely accepted theory in the scientific community, while the tau protein hypothesis is the least widely accepted theory in the scientific community, according to the A*β* hypothesis. Amyloid precursor proteins accumulate in the brain after being broken down by the enzyme *β*-secretase, resulting in the production of amyloid *β*-plaques, which then lead to the accumulation of A*β* protein. As a result of this form of cutting, several distinct chemicals are created, each of which has the potential to play a role in the progression of AD over time. On the other hand, the molecule A*β* is the most important of the three components. Despite its high proclivity for incorrect folding, it accumulates and forms oligomers, which aggregate in the brain and eventually form plaques, resulting in a loss of nervous system function. Using currently known methodologies, distinguishing between the various types of oligomers that have the potential to be harmful to human health is difficult. Cerebral amyloid vascular disease (CAVD), also known as cerebral amyloidosis, is characterized by the formation of neurotic plaques from extracellular A*β*. Diffuse A*β* plaques were seen in the frontal and parietal lobes of the brain early in the disease's course, confirming the virus's existence. The exact course of the disease is unknown at this time; however, as the disease progresses, diffuse plaques and neurogenic plaques are expected to become more prevalent throughout the wider neocortical region, spreading in the following directions: neocortex, hippocampus, brain stem, and cerebellum. Another theory holds that the tau protein is responsible for reducing A*β*'s impact on brain function. The tau protein seen in AD patients' brains is frequently phosphorylated and disorganized, which is also true in healthy people, according to the same findings in the general population. Because tau protein is insoluble, it clumps together and takes on a variety of shapes and forms, making it extremely hazardous [13]. The pathogenic tau protein can misfold and spread throughout the brain because of interactions between it and healthy neurons in the nearby area, resulting in AD and other neurodegenerative illnesses such as Parkinson's disease and vascular dementia. These proteins are known as prion-like proteins in the scientific community because they can induce the same aberrant conformation in homologous proteins [14], a trait that renders them contagious. Because homologous proteins tend to generate the same abnormal conformation, a self-amplification cascade begins at some point throughout the process. A causal link between A*β* and the production of p-tau has been established by many investigations [15, 16], and these findings have been verified by other studies. The axonal protein tau, as well as its phosphorylated form (p-tau), is involved in the postsynaptic targeting of the Src kinase Fyn, which has the neurotransmitter NMDA as a substrate. An animal study has revealed that tau has a dendritic influence on A*β*, resulting in postsynaptic toxicity of A*β* in transgenic mice expressing human amyloid precursor protein, and that lowering endogenous tau levels in these transgenic mice improves the transgenic mouse's behavioral issues. Oligomeric A*β* was also discovered to stimulate astrocytes to release glutamate, which was then followed by synaptic NMDA receptor activation, which led to increased tau levels in brain neurons by Roberson and colleagues [16]. Every one of these research supports the hypothesis that, as previously stated, the presence of tau in the environment regulates A*β* toxicity. The amount of neurotoxicity present in the body, rather than the amount of A overexpressed or depleted in the body, is of special concern [17]. A spike in p-tau expression has been linked to a reduction in the overall number of synaptic connections in some studies; however, this has not always been the case. The protein tau grows abnormally in nerve cells and glial cells due to a shortage of microtubules. Several neurodegenerative diseases are thought to be caused by this aberrant development. Researchers have just discovered the A*β* protein called p-tau, which has been linked to microtubules for a long time. Proteins with a lot of p-tau cannot connect to microtubules, just like normal proteins (as shown in Figure 1) [18]. Tau inactivity causes a failure to drive the formation of microtubules, resulting in the cellular structures' disintegration [19]. Tau is made up of a filamentous bundle of nerve fibers that gives it its name and gives it its distinctive shape. According to the findings, the protein tau has been shown to have the ability to block the development of neurons in laboratory trials.

Furthermore, neuroinflammation has been implicated as a factor in AD and other diseases [19, 20]. The formation and advancement of glial cell types, the most important of which are microglia and astrocytes, are a distinguishing hallmark of AD. They are also believed to be involved in the disease's etiology. These cells are known as immune effector cells of the central nervous system (CNS) because of their ability to communicate with the immune system, and they play a critical part in the body's response to infection and other disorders. They are glial cells in the central nervous system that may be counted on one hand as the most abundant. They can also fine tune the environment while providing nutritional and metabolic support for neurons [20, 21]. The ability to modify a variety of physiological parameters, such as pH, ion balance, oxidative stress, and blood flow, is another aspect of these devices. Glacial cells are responsible for a wide range of environmental tasks when they are active. In the event of a brain injury, one of these roles is to protect the brain by responding as rapidly as possible. On the other hand, uncontrolled and chronic stimulation would have the opposite effect on the brain's ability to operate effectively. When microglia meet this environment, they suffer apoptosis, which transforms them into cells with proinflammatory characteristics. Neuron death is caused by inflammatory chemicals, ROS, and nitric oxide, which are produced by the body and cause neuron death. Numerous studies have been conducted on tumor necrosis factor (TNF), also known as an inflammatory factor, to better understand how it functions and affects the body. The inflammatory mediator TNF interacts with local cells through the tumor necrosis factor receptor as it travels to and from the ventricle, starting a cascade that results in increased TNF production [22, 23]. The nuclear factor kappa light chain enhancer of activated B cells activates the NF-*κ*B pathway, which produces TNF [22, 23]. TNF inhibits phosphoinositide 3-kinases (PI3KS) and mitogen-activated protein kinases (MAPKs) and hence increases the activity of glycogen synthase kinase 3 (GSK-3) in the brain (MAPK). Increased GSK-3 activity can lead to the formation of amyloid plaques in the cerebrovascular system. TNF-*α* has been shown to stimulate phosphorylation of active protein 1 in the presence of a catalyst [23, 24], a process that has been connected to p-tau [23, 24]. The inflammatory response has also been connected to TNF-*α* [23, 24]. According to studies, TNF has been demonstrated to boost the phosphorylation of active protein 1 by a factor of two when combined with a catalyst. A laboratory study has shown that when TNF-*α* is used in conjunction with a catalyst, it enhances the phosphorylation of active protein 1 in the presence of the catalyst. Amyloid can promote mitochondrial malfunction and astrocyte death in astrocytes by increasing the generation of ROS in the cells, according to new research [25]. After it was revealed that amyloid can activate NADPH oxidase in astrocytes by increasing the formation of ROS in the cells, this was discovered. This discovery was made due to an increase in the generation of ROS in the cells. The link between the CNS inflammatory response and amyloid protein is widely believed by experts [26] to be at the root of AD.

**Figure 3:**
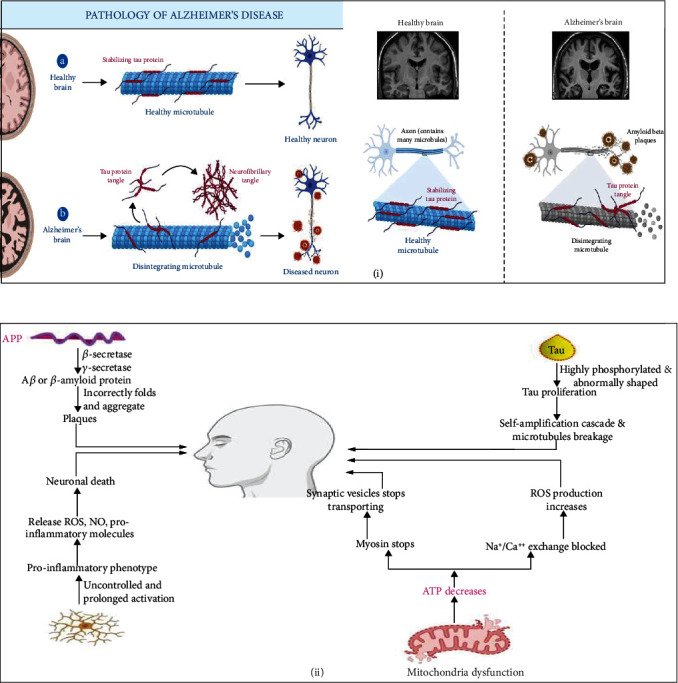
(a) Pathology and (b) pathophysiology of AD (made with BioRender).

Mitochondria (also known as mitochondrial organelles) are mushroom-like microorganisms that play a key role in cell division. They can be found in all cells and play a crucial role in the formation of new ones. Mitochondria can be found in all cells and organs, and they are essential for the survival of life on Earth. A growing number of researchers have found that mitochondrial dysfunction is a crucial factor in the onset of AD [27, 28], some of which employed postmortem brain samples from AD patients and others that used laboratory models. Mitochondrial dysfunction is more common in patients who are in the early stages of the disease, according to several studies. During this intricate process, several critical functions are carried out, including maintaining adequate intracellular calcium homeostasis, maintaining an ideal intracellular redox equilibrium, and mediating apoptosis and necrosis [29]. The mitochondrial function includes maintaining correct intracellular calcium balance, regulating intracellular redox balance, and regulating intracellular redox balance, in addition to mediating apoptosis. Mitochondrial activity is intricately linked to a variety of essential functions, including regulating intracellular redox homeostasis and mediating apoptosis. Because mitochondria are such an important source of energy, when they malfunction, the amount of ATP produced decreases, with synaptic structural damage serving as the first sign that something is amiss. Because the synaptic structure of the neuron is so vital for efficient neurotransmission in a cell, the neuron itself must be in good working order for the transmission to be effective. It has been proven that synaptic vesicles are unable to transport when there are no ATP-dependent neurotransmitters to release or when the amount of ATP required for the requisite myosin labor is insufficient [30]. This is due to a shortage of ATP availability. According to some experts, cognitive impairment is caused by a synthetic deficiency of acetylcholine (ACh), a neurotransmitter important for memory and learning [31]. The cholinergic hypothesis was the first and most completely investigated concept when it came to understanding the pathophysiology of AD on a cellular level, and it remains the most widely accepted to this day. Downregulation of cholinergic markers such as acetylcholinesterase, a cholinergic enzyme, occurs because of this distinct shift in cholinergic activity, which is also visible in the brain. According to recent research [32], alterations in cholinergic markers, the density of aberrant nerve fibers, and the severity of the illness have a proportional relationship. Synaptic transmission and plasticity problems, for example, are common in AD, which is marked by memory loss and other cognitive abnormalities. Synaptic plasticity issues, as well as a loss in synaptic density and a reduction in synaptic transmission, are all prevalent signs of AD, and they are all linked to the disease. Mitochondria oversee two important functions: keeping calcium concentrations at a constant level and calcium homeostasis, both of which are essential for the cell's normal functioning. Both of these processes are required for the cell to function properly. If ATP levels drop, the amount of energy available for the sodium-citrate-citrate-citrate exchange pathway, which is critical for calcium removal from neuronal cells, may also drop. A previous study found that neurotoxicity could be caused by too much ROS formation if the situation is not controlled [33]. The last thing on the list, oxidative phosphorylation of cellular components, needs mitochondria to work because it needs oxygen. A lack of ATP in the brain is one of the most apparent signs of AD, linked to mitochondrial OXPHOS failure [34]. According to research [35], researchers discovered that the activity of mitochondrial complex IV in platelets from AD patients has decreased. Fang's research proved that there is a previously unknown association between PTEN-induced decreased expression of presumed kinase 1 (PINK1) and AD etiology. Following gene therapy-mediated PINK1 overexpression, increased activation of the autophagy receptor (OPTN NDP52) in AD mice achieves the goal of cleaning damaged mitochondria by reducing synaptic loss caused by amyloid protein and cognitive decline in AD mice [36]. Following gene therapy-mediated PINK1 overexpression, increased activation of the autophagy receptor (OPTN NDP52) in AD mice fulfills the goal. Autophagy vacuoles have recently been discovered to contain both the A-*β* protein and the secretase enzyme, which are both essential for protein production. The findings imply that the A*β* protein is an important component of the *β*-secretase complex, with specialized activity in the *β*-secretase domain. According to a new study, mitochondria play a critical role in the regulation of cell death processes. Even though additional research is needed to fully understand the link between mitochondria, autophagy, and AD, this is a significant advancement in the field of neurodegenerative disease. The pathophysiology and pathogenesis of AD were shown in Figure 3.

## 3. Pharmacological Effects of Asian and South-Asian Traditional Plants on AD

### 3.1. *Curcuma longa*

#### 3.1.1. The Plant

It is the botanical name given to the ginger plant *Curcuma longa*, which belongs to the Zingiberaceae family and has the scientific name *Curcuma*. Turmeric is a sterile plant, which means that it produces no seeds. The plant can grow to a height of 3–5 feet and has dull yellow blossoms on the top of its branches. The subterranean rhizomes or roots of the plant are used for a variety of medicinal and culinary applications. A subterranean stem that is thick and meaty and encircled by the roots of ancient leaves is known as a rhizome. Turmeric is created by boiling rhizomes, drying them, and grinding them into a bright-yellow spice [37]. Turmeric is derived from the root turmeric.

### 3.2. Effects of *Curcuma longa* on AD

**Figure 4:**
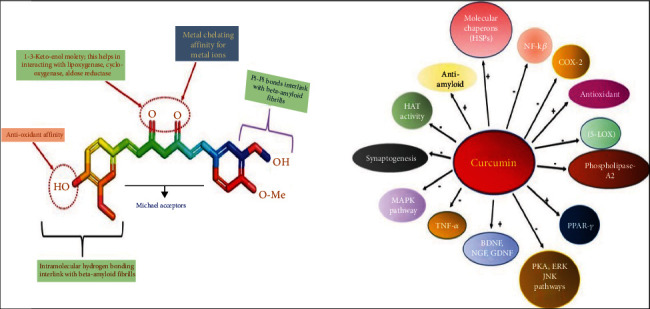
Curcumin's structure indicates functional groups that could be effective in AAD disease treatment. Curcumin can be used to treat neurodegenerative diseases for a variety of reasons (made with BioRender).

AD is characterized by chronic nerve cell inflammation, which is one of the key contributing factors. Microgliosis, astrocytosis, and the presence of proinflammatory chemicals have all been linked to the deposition of A*β* peptides in several studies [38]. Curcumin lowers inflammation by inhibiting the early growth response-1 (Egr-1) protein's ability to bind to DNA. Curcumin has been demonstrated to inhibit monocyte chemotaxis, a process triggered by chemokines generated by active microglia and astrocytes in the CNS [39, 40]. Cyclooxygenase-2(COX-2), phospholipases, transcription factors, and enzymes involved in the conversion of membrane phospholipids to prostaglandins have all been shown to be inhibited by curcumin [41]. COX-1, COX-2, and COX-3 have all been demonstrated to be inhibited by curcumin. Reduced formation of ROS by activated neutrophils and suppressed expression of the proinflammatory cytokines TNF-*α* and IL-1*β* are all efficient approaches to prevent proinflammatory cytokine activation [42, 43]. Curcumin works by inhibiting the transcription factor AP-1, which is involved in the development of amyloid, a protein linked to AD. Curcuminoids have been shown in tests to have strong antioxidant properties, as evidenced by their ability to inhibit free radical formation and propagation. This chemical inhibits the oxidation of low-density lipoproteins and free radicals, which cause neuron degeneration in AD and other neurodegenerative disorders like Huntington's and Parkinson's disease (PD) [43]. A single dose of curcumin (1 and 2 mg/kg, intravenously) given after focal cerebral ischemia/reperfusion in rats reduced infarct volume and improved neurological impairment while also lowering mortality and decreasing the brain water content, according to researchers at Nanjing Medical University (China) [44]. Researchers discovered that curcumin administration reduced lipid peroxidation and lipofuscin accumulation, both of which are generally related to aging [45]. Jawaharlal Nehru University in India conducted the research. Curcumin boosts heme oxygenase activity by encouraging the inactivation of the nuclear factor erythroid 2-related factor 2- (Nrf2-) keap1 complex and enhancing no-1ARE binding to the enzyme. Astrocytes revealed a large increase in reduced glutathione levels first, followed by a considerable increase in oxidized glutathione levels after incubation with curcumin at a concentration that increased heme oxygenase activity [46–48]. Curcumin's structure indicates functional groups that could be effective in AAD disease treatment shown in Figure 3. The most visible symptom of AD is the appearance of *β*-amyloid plaques. The levels of *β*-amyloid in AD animals given low doses of curcumin were reduced by roughly 40% compared to those who did not get curcumin in contrast to those who did not receive curcumin. Curcumin at low doses was also reported to reduce the “plaque burden” of *β*-amyloid in AD mice's brains by 43% [49]. Curcumin may be used to treat neurodegenerative diseases for a variety of reasons shown in Figure 4.

### 3.3. *Withania somnifera*

#### 3.3.1. The Plant

The nightshade (Solanaceae) family includes ashwagandha. It is classified as a Rasayana (rejuvenating) and is said to have antioxidant properties, free radical scavenging properties, and the capacity to maintain a healthy immune system [50]. Twelve alkaloids, 40 with anolides, and many sitoindosides and flavonoids are among the chemicals found in ashwagandha root [51–53].

#### 3.3.2. Effects of *Withania somnifera* on AD

**Figure 5:**
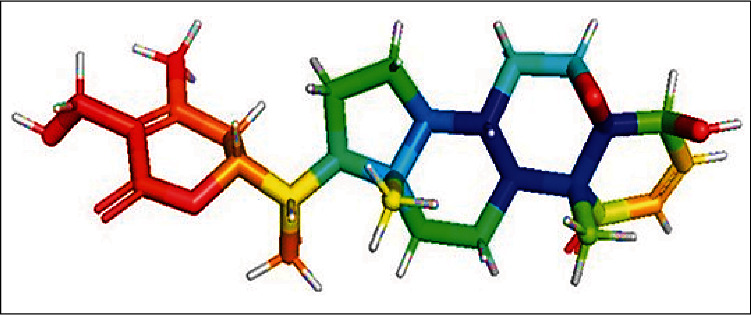
Molecular structure of *Withania somnifera.*

Ashwagandha root, according to a study conducted at the molecular level, may be beneficial in the treatment of AD by inhibiting nuclear factor-B activation, inhibiting *β*-amyloid (A*β*) production, decreasing apoptotic cell death, restoring synaptic function, and increasing antioxidant effects by promoting the migration of Nrf2 to the nucleus, where it increases antioxidant enzyme expression [54]. The treatment of human neuroblastoma SK-N-SH cells with methanolic preparations of ashwagandha root resulted in an increase in dendritic extension, neurite outgrowth, and synapse formation, according to the findings [55]. A (25–35) (10 M) treatment in cultured rat cortical neurons causes axonal and dendritic shrinkage, as well as pre- and postsynaptic loss, according to the findings [56]. These alterations are reversed by treatment with WL-A (1 M), the researchers discovered. Aside from that, WL-A inhibits the expression of semaphorin 3A, which allows for more straightforward neuronal regeneration. According to some researchers, the beneficial effects of ashwagandha root constituents on neurodegenerative diseases may be due to their neurite-promoting, anti-inflammatory, antiapoptotic, and anxiolytic properties. Additionally, their ability to alleviate mitochondrial dysfunction and restore energy levels, as well as their ability to raise levels of antioxidant defenses, such as reduced glutathione [57], may account for their effectiveness. Although some people feel diarrhea or nausea after ingesting the root, ashwagandha is a completely safe herb to consume and consume regularly. When combined with barbiturate-type sedatives, it has the potential to improve their effectiveness; hence, it should not be used in conjunction with them. According to several studies, ashwagandha can pass the blood-brain barrier and decrease inflammation in the brain [58, 59]. The molecular structure of *Withania somnifera* was shown in Figure 5.

### 3.4. *Convolvulus pluricaulis*

#### 3.4.1. The Plant

*Convolvulus pluricaulis* is a member of the Convolvulaceae family. It is used to help people remember things. A recent study found that *Convolvulus pluricaulis* aqueous and ethyl acetate extract improve memory and learning ability [60].

#### 3.4.2. Effects of *Convolvulus pluricaulis* on AD

**Figure 6:**
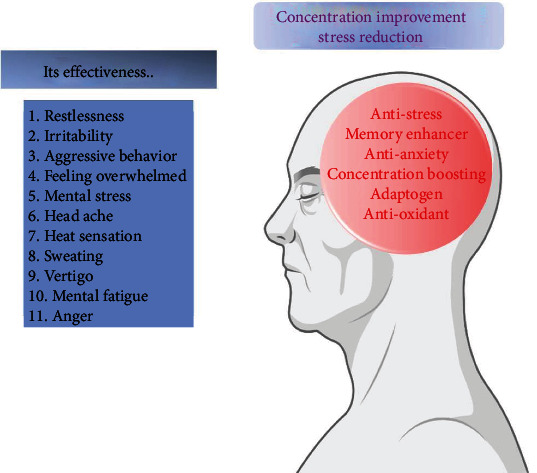
Effectiveness of *Convolvulus pluricaulis* on different mental diseases (made with BioRender).

Several types of stress are relieved by the plant, including psychological, chemical, and traumatic stress. The roots of CP have been shown to reduce total serum cholesterol, triglycerides, and phospholipids while increasing brain protein concentration [61]. CP is a well-known Ayurvedic plant that is noted for its effectiveness in the treatment of anxiety and neurosis. The phytochemical study has revealed the occurrence of alkaloids (Shankhapushpine), triterpenoids, flavonoids, glycosides, anthocyanins, and steroids. The pharmacological activity of these metabolites is diverse, including nootropic and memory-enhancing effects [62, 63]. The effectiveness of *Convolvulus pluricaulis* on the different mental diseases was shown in Figure 6.

The ability of the plant to inhibit the accumulation of lipid and protein damage further demonstrated its neuroprotective potential. In addition, when compared to the standard AD treatment rivastigmine, CP extract administration reduced changes in endogenous antioxidant enzyme levels related to Al administration [64]. The ethanolic extract of CP exhibits significant antioxidant activity, according to in vitro research [65–67].

### 3.5. *Centella asiatica*

#### 3.5.1. The Plant

*Centella asiatica*, sometimes known as Asiatic pennywort or gotu kola, is a plant that has been utilized as an alternative to AChEI. It is a perennial herbaceous creeper with kidney-shaped leaves that is popular in Asian countries [68, 69]. This plant is used as a spice, a vegetable, and a juice and in nutraceutical and cosmetic products. For its antipyretic and wound-healing capabilities, *Centella asiatica* has been included in Thailand's National List of Essential Medicines [70].

#### 3.5.2. Effects of *Centella asiatica* on AD

**Figure 7:**
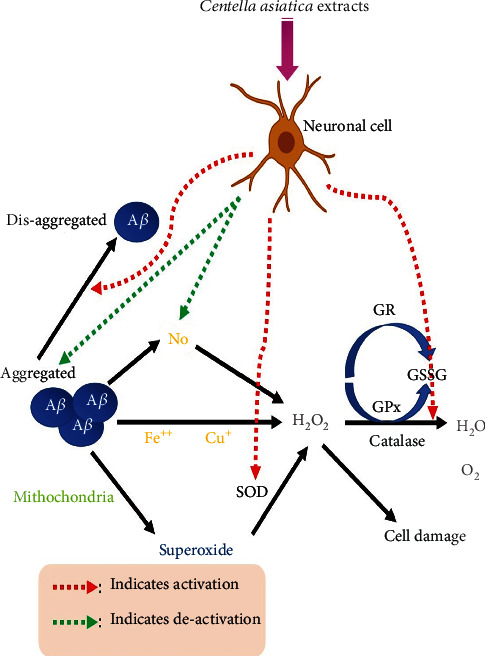
*Centella asiatica* extract activates the antioxidative defense system in neuronal cells, protecting them from amyloid 1–40-induced neurotoxicity (made with BioRender).

*Centella asiatica*'s effects on neuronal morphology, learning performance, and memory retention have all been demonstrated in animal studies [71, 72]. It has been demonstrated to increase cognitive function by inhibiting acetylcholinesterase activity, lowering phospholipase A2 (PLA2) activity, protecting against *β*-amyloid production, and protecting against brain injury [73–75]. *Centella asiatica* has also been found to have antistress, antidepressant, anxiolytic, and seizure-preventing activities in preclinical trials [76–78]. *Centella asiatica* has also been shown in preclinical trials to have depressive, antidepressant, anxiolytic, and seizure-preventing effects [76–78]. Asiaticoside and Asiatic acid have been found to exhibit neuroprotective, antidepressive, and anxiolytic effects in animal models [79–82]. Asiaticoside and Asiatic acid have been discovered to exhibit neuroprotective, antidepressive, and anxiolytic activities in animal experiments [81, 82]. Asiatic acid has been shown to improve learning and memory using both passive and active avoidance tests [83]. *Centella asiatica* has been shown to diminish *β*-amyloid accumulation in the 5xFAD animal model of AD. *Centella asiatica* has been demonstrated to impact Alzheimer's disease-related metabolic pathways when given to the 5xFAD mice model of *β*-amyloid accumulation. *Centella asiatica* has been shown to improve memory and executive function while reducing hippocampal mitochondrial dysfunction in rats that have been overexpressed with *β*-amyloid [83]. *Centella asiatica* extract activates the antioxidative defense system shown in Figure 7.

### 3.6. *Celastrus paniculatus*

#### 3.6.1. The Plant

Malkangani (*Celastrus paniculatus* Willd.) is a woody climber and a frequently used medicinal herb in the Unani Medicare System. It belongs to the Celastraceae family. It may reach a height of 18 meters and has a stem diameter of 23 centimeters.

#### 3.6.2. Effects of *Celastrus paniculatus* on AD

**Figure 8:**
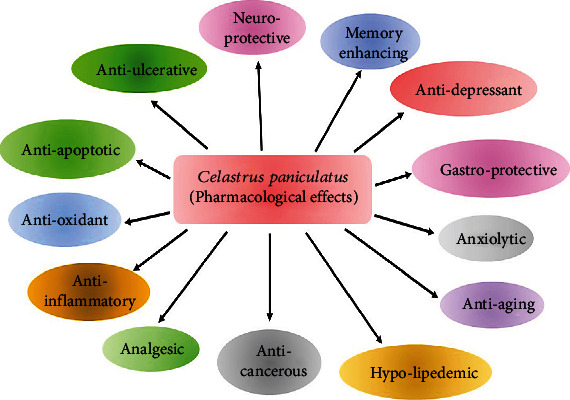
Pharmacological effects of *Celastrus paniculatus* (made with BioRender).

Several studies have looked into the neuroprotective properties of *Celastrus paniculatus*, as well as other herbs such as *Centella asiatica* and *Curcuma longa* [84]. Glutamate-induced neuronal injury was prevented in the primary forebrain culture by *Celastrus paniculatus* therapy, according to Godkar and colleagues [85, 86]. *Celastrus paniculatus* treatment reduced NMDA receptor-driven whole-cell currents, according to the researchers. Two weeks of *Celastrus paniculatus* therapy protected rats from the neurotoxicity caused by kainic acid in the lab. Treatment resulted in behavioral improvement in the shuttle box and Morris water maze tests, according to the findings. Both corticosterone and cholinesterase levels were found to be increased in the bloodstream. An antiepileptic effect was observed on mice when a methanolic extract of the entire *Celastrus paniculatus* plant was administered. It was discovered that both isoniazid-*β*- and pentylenetetrazole-induced epileptic seizures were reduced after *Celastrus paniculatus* treatment [87]. By research [88, 89], cholinergic neurotransmission is critical in the establishment of long-term memory [90]. Scopolamine, a muscarinic cholinergic receptor antagonist, has been shown to impair memory and learning in humans via changing cholinergic neurotransmission in the brain [91]. The use of *Celastrus paniculatus* therapy in rats was shown to improve spatial navigational memory deficiencies caused by scopolamine in a Morris water maze challenge [92]. The pharmacological effects of *Celastrus paniculatus* were shown in Figure 8.

### 3.7. *Coriandrum sativum*

#### 3.7.1. The Plant

*Coriandrum sativum* is a member of the Apiaceae family; the annual herb *Coriandrum sativum* comes from the Mediterranean region. The leaves are little branches and subbranches, the blooms are white, and the fruits are nearly ovate globular in shape with many longitudinal ridges on the surface [93].

#### 3.7.2. Effects of *Coriandrum sativum* on AD

In one study, male Wistar rats were administered *Coriandrum sativum* for 45 days to see if it improved their cognitive ability. This study compared aging, scopolamine, and diazepam-induced forgetfulness. Because of its antioxidant, anti-inflammatory, and cholesterol-lowering properties, *Coriandrum sativum* improved memory [94]. In Iranian folk medicine, *Coriandrum sativum* has been used to treat sleeplessness [95, 96]. Presleep use of a single dose of chopped fresh leaves (30 g) or seeds of the plant with tea has been shown to alleviate anxiety and insomnia [95]. Animal models were used to confirm these effects. In male albino mice, intraperitoneal (i.p.) treatment of aqueous extracts (200, 400, and 600 mg/kg), as well as 400 and 600 mg/kg EO of coriander, enhanced the sleep duration to 160–220, 130–180, and 210 minutes, respectively. These findings revealed that coriander extracts and essential oils have sedative and hypnotic properties in a pentobarbital-induced hypnotic paradigm [97]. Another study found that 10 ml/kg (i.p.) of a fresh plant extract reduced immobility time better than a dried plant extract. The extract of the green plant, but not the dried plant, was found to exhibit antidepressant properties [98]. The hydroalcoholic extract of *C. sativum* (50, 100, and 200 mg/kg) reduced the duration, frequency, and amplitude of burst discharges while also prolonging seizure attack latency. In different parts of the hippocampus, the extract also stopped the formation of dark neurons and apoptotic cells [99].

### 3.8. *Ficus carica*

#### 3.8.1. The Plant

The fig tree and its fruit, known as the common fig, are commonly referred to as Ficus (*Ficus carica*). The common fig is a big deciduous shrub or small tree that is native to Southern Asia and the Eastern Mediterranean (Greece, east to Afghanistan). With smooth grey bark, it grows to a height of 3–10 m. Throughout its natural area in Iran, as well as the rest of the Mediterranean region, the common fig is widely farmed for its edible fruit [100].

#### 3.8.2. Effects of *Ficus carica* on AD

*Ficus carica* has a minor memory-enhancing effect at low doses, while at greater levels, it improves learning ability and changes behavior. Because of its antioxidant action, *Ficus carica* includes quercetin, which plays a key role in memory loss and AD. Mice with memory deficits and normal mice were employed in this study. The efficiency of *Ficus carica* on cognitive processes was evaluated using a rectangular maze model and a *Y*-maze [101]. The three fruit portions showed significant differences in bioactivity against acrolein radicals in a concentration-dependent experiment, and metabolites from the mesocarp and endocarp showed bioactivity in the scavenging of ROS radicals. NMR profiling revealed that aliphatic chemicals like sitosterol tend to generate neuronal bioactivity and show bioactivity in a cell survival assay. *γ*-Sitosterol was identified in higher concentrations in the mesocarp of *Ficus carica* and was deemed a significant phytosterol [102].

### 3.9. *Magnolia officinalis*

#### 3.9.1. The Plant

*Magnolia officinalis* is a member of the Magnoliaceae family. It helps the memory problems caused by scopolamine [103].

#### 3.9.2. Effects of *Magnolia officinalis* on AD

Magnolia officinalis decreases the action of acetylcholinesterase [104]. Its ethanolic extract containing honokiol and magnolol has been shown to have antioxidant properties [105–107].

Various Soxhlet and supercritical fluid extracts have been shown to have antioxidant activity in vitro, with the ethyl acetate Soxhlet extract being the most potent [108]. Magnolol has been shown to have neuroprotective properties in vitro [109]. Honokiol inhibits the production of reactive oxygen species, which has anti-inflammatory properties [110]. *Magnolia officinalis* is useful in the treatment of AD and memory loss since it is anti-inflammatory and antioxidant [111–113].

### 3.10. *Myristica fragrans*

#### 3.10.1. The Plant

*Myristica fragrans* is an evergreen tree native to Indonesia's the Maluku Islands. Guangdong and Yunnan in China, Taiwan, Indonesia, Malaysia, Grenada in the Caribbean, Kerala in India, Sri Lanka, and South America are just a few of the places where it is grown [114]. *Myristica fragrans* is a member of the Myristicaceae family [115].

#### 3.10.2. Effects on *Myristica fragrans* AD

*Myristica fragrans* includes camphene, b-pinene, sabinene, cymene, garaniol, d-borneol, linolool, terpineol, safrol, elemicin, myristicins, phenylpropane derivatives, lauric acid, myristic acid, pentadecanoic acid, palmitic acid, and heptadecanoic [115]. Nervous diseases, digestive disorders, leukemia, body ache, vomiting, tachycardia, dizziness, and memory impairments are all treated with *Myristica fragrans* [116]. It has antidepressant, antioxidant, and antibacterial properties [117]. Young and old mice were given three doses of N-hexane extract of *Myristica fragrans* (5, 10, and 20 mg/kg p.o.) orally over three days. At 5 mg/kg, this medication was found to help correct the learning and memory deficits caused by scopolamine and diazepam. The utility of *Myristica fragrans* in treating AD and memory problems has been proven in this study [118].

### 3.11. *Bacopa monnieri*

#### 3.11.1. The Plant

The Scrophulariaceae family includes *Bacopa monnieri.* It is a creeping perennial herb native to the marshes of India's southern and eastern regions, as well as Australia, Europe, Africa, Asia, and North and South America [119].

#### 3.11.2. Effects of *Bacopa monnieri* on AD

Sterols, saponins, alkaloids, Monnier, saponin acid A, herpes time, and brahmins are all found in *Bacopa monnieri* [119]. *Bacopa monnieri*, in combination with *Centella astiatica* and *Evolulus alsinoides*, is used by traditional healers to treat memory impairments and AD [120]. It improves memory in ADA sufferers. It is an adaptogenic, neuroprotective, antibacterial, and memory booster all rolled into one [121]. Calabrese and his colleagues [122] investigated the effects of *Bacopa monnieri* on cognitive performance, anxiety, and depression in the elderly and discovered that it effectively improved cognitive functioning. This research backs up its use as a memory booster. In a rat model of AD, another study found that *Bacopa monnieri* prevents cholinergic degeneration and improves cognition [123].

#### 3.11.3. Common Habitat Areas

Table 1 shows the natural habitat areas of the previously discussed medicinal plant.

## 4. Natural Product Distribution to the Brain: Challenges and Innovative Delivery Technologies

In part, the presence of physical barriers in the brain, such as the blood-brain barrier, makes treating AD more difficult than it should be a blood-brain barrier (BBB). Biological barriers are critical barriers that must be crossed for naturally occurring chemicals to be delivered into the brain from the bloodstream. While studying the activity of neuroprotective natural substances, it is important to note that the difficulties posed by the BBB are significant because their efficiency is significantly reduced by their limited bioavailability and occasionally by their poor pharmacokinetic profile.

Many anti-inflammatory natural chemicals are assumed to be bioavailable because of their metabolism, which is thought to be responsible for this. Its anti-inflammatory and antioxidant capabilities have been thoroughly explored, and curcumin is a polyphenol that possesses both qualities. Despite this, the molecule's pharmacological potential is limited due to the low bioavailability of the substance after oral administration. In addition to its antioxidant and anti-inflammatory capabilities, research has demonstrated that resveratrol may also have neuroprotective benefits. Because of the low bioavailability properties of these polyphenols, researchers have been striving to generate more bioavailable derivatives of these compounds for several years.

A variety of delivery techniques, most of which used lipid-based nanocarriers, have been used to investigate the bioavailability and BBB penetration of natural substances that are neuroprotective. Using the example of liposomes, which are produced by amphiphilic substances such as phospholipids that self-assemble into vesicles consisting of lipid bilayers and are then released into the environment, we can see how they work. Lignin nanoparticles have gained in popularity over the years, and they have the added benefit of protecting active compounds from degradation while they are in use. A new class of submicron-sized lipid emulsions has emerged in which the liquid lipid (oil) has been substituted with a solubilized lipid that can penetrate the blood-brain barrier and exert a pharmacological effect on the central nervous system, known as soluble lipid nanoparticles (SLNs) [131].

## 5. Conclusion and Future Directions

According to the American Diabetes Association, AD is a debilitating ailment that has had a substantial negative impact on modern society. Medications to prevent or treat AD are continuously being developed. Symptoms can only be treated with the usage of existing substances. As the average life expectancy rises, the discovery and development of new compounds capable of preventing and treating AD are becoming increasingly crucial. A variety of natural substances have shown promise as AD treatments in clinical and preclinical studies. Clinical experiments have revealed that some chemicals appear to be effective in treating AD, whereas others have failed in human testing. More research is needed to establish whether natural compounds have therapeutic promise for AD.

In the case of neurodegenerative illnesses, natural products have demonstrated promising health-promoting effects, which can be attributed in part to their anti-inflammatory properties. For this reason, as the number of people suffering from AD continues to rise, new study findings on the possible therapeutic effects of natural compounds **o**n the treatment of the condition are very hopeful.

The potential for anti-inflammatory natural compounds to treat AD has been demonstrated in numerous investigations; however, additional research is required to evaluate their clinical usefulness in well-controlled human studies. The findings of in vitro efficacy trials, according to some research, may not necessarily translate into in vivo benefits. It is also not feasible to extrapolate results from animal models to individuals seeking new Alzheimer's treatments because of major species variances. To solve this issue, preclinical research on anti-inflammatory natural compounds must emphasize the use of cutting-edge technology in addition to traditional approaches. One of these approaches is the in vitro study of neuroinflammation and neuroprotection using a triculture of human neurons, astrocytes, and microglia to examine the effects of various drugs on the brain.

**Figure 9:**
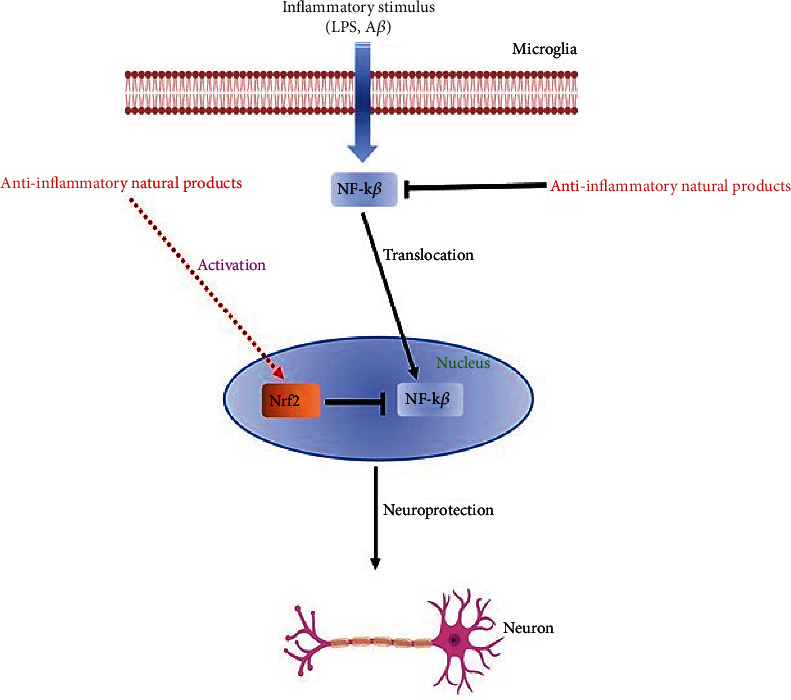
The transcription factors NF-*κ*B and Nrf2 are among the molecular targets of anti-neuroinflammatory natural product action (made with BioRender).

Small molecules for AD must be accessible to the brain and capable of crossing the blood-brain barrier to be effective in the disease. It has been demonstrated in published studies that nanocarriers could transfer natural compounds to the cerebral cortex. A greater emphasis should be placed on the development of innovative delivery systems for polar phytochemicals (such as anti-inflammatory/antioxidant polyphenols) to achieve high therapeutic concentrations in the brain. The transcription factors NF-*κ*B and Nrf2 are among the molecular targets of anti-neuroinflammatory natural product action shown in Figure 9.

Because of the complex pathways involved in AD, concentrating on a single gene for molecular target-driven identification of novel natural products for the disease is not the most appropriate disease model. Most pharmacologically active natural compounds found in this manner have not resulted in the development of new therapies. The primary focus of research should be on the two most important transcription factors, NF-*κ*B and Nrf2, which control critical molecular roles in the generation of inflammation or the suppression of anti-inflammatory responses. A further recommendation is that preclinical research into AD is conducted using animal models that involve stress reduction (neuroinflammation and oxidative stress), neuroprotection, and regeneration.

## Figures and Tables

**Table 1:** The natural habitat areas of plants.

Name of the plant	Habitat area	Treatments in AD	References
*Curcuma longa*	India, China, Pakistan, Ghana, Kenya, and Nigeria	Curcumin maintains the normal structure and function of brain arteries, mitochondria, and synapses, reduces chronic disease risk factors, and reduces Alzheimer's disease risk	[124]
*Withania somnifera*	The Middle East and Arabia, Afghanistan, Baluchistan, Pakistan, Sri Lanka, China, Nepal, and India	Liver upregulation LRP shows that targeting the periphery clears A*β* and reverses Alzheimer's disease pathogenesis	[125]
*Convolvulus pluricaulis*	India and Burma	*C. pluricaulis* extract (aqueous) protects Alzheimer's disease (AD) Drosophila model against hMAP-induced neurotoxicity	[126]
*Centella asiatica*	Tropical and subtropical regions of India, Southeast Asia, and Malaysia, as well as some temperate regions of China, Korea, Japan, and Taiwan	Have significant antioxidant activity that reduces brain A*β* deposition. Aggregated A*β*-induced oxidative stress triggers Alzheimer's disease (AD)	[127]
*Celastrus paniculatus*	China and Southeast Asia	The antioxidant and anti-Alzheimer activities of the crude methanolic extract of *Celastrus paniculatus* seeds were investigated. The extracts scavenged DPPH free radicals, inhibited peroxynitrite (ONOO−), and reduced ROS production	[128]
*Coriandrum sativum*	In the tropical and subtropical region, Pakistan, and all South-East Asian countries		[129]
*Ficus carica*	South Asian to Easter pedestrian	Mesocarp and endocarp metabolites scavenged ROS. NMR profiling showed that aliphatic chemicals like *γ*-sitosterol enhance neuronal bioactivity and cell viability. *γ*-Sitosterol is a significant phytosterol in *Ficus carica*'s mesocarp	[100]
*Magnolia officinalis*	Mainly in China	*Magnolia officinalis* ethanol extract and 4-O-methylhonokiol suppress AChE, preventing scopolamine-induced memory loss	[130]
*Myristica fragrans*	Indonesia's Maluku Islands, Guangdong and Yunnan in China, Taiwan, Indonesia, Malaysia, Grenada in the Caribbean, Kerala in India, and Sri Lanka	*M. fragrans* showed anticholinesterase, *α*-glucosidase inhibitory, and antioxidant effects in vitro, making it a viable Alzheimer's disease treatment	[114]
*Bacopa monnieri*	India's southern and eastern regions, as well as Australia, Europe, Africa, Asia, and North and South America	It decreases *β*-amyloid and stress-induced hippocampus damage. EBm's neuroprotective action is mediated via nitric oxide. In humans, EBm increased logical memory and paired association learning and reversed phenytoin-induced memory impairment	[119]

## Data Availability

The data supporting the findings of this study are available within the article.

## Abbreviations

TCM: Traditional Chinese Medicine
AD: Alzheimer's disease
A*β*: Amyloid-*β*
ROS: Reactive oxygen species
NF-*κ*B: Nuclear factor-kappa B
NFTs: Neurofibrillary tangles
CDC: Centers for Disease Control and Prevention
FDA: Food and Drug Administration
CAVD: Cerebral amyloid vascular disease
CNS: Central nervous system
TNF: Tumor necrosis factor
PI3KS: Phosphoinositide 3-kinases
MAPKs: Mitogen-activated protein kinases
GSK-3: Glycogen synthase kinase 3
ACh: Acetylcholine
PINK1: PTEN-induced decreased expression of presumed kinase 1
Egr-1: Early growth response-1
COX-2: Cyclooxygenase
PD: Parkinson's disease
Nrf2: Nuclear factor erythroid 2-related factor 2
BBB: Blood-brain barrier
DDS: Drug delivery system
SLNs: Soluble lipid nanoparticles
NMR: Nuclear magnetic resonance.
